# Comparison of the Biological Characteristics of Mesenchymal Stem Cells Derived from the Human Placenta and Umbilical Cord

**DOI:** 10.1038/s41598-018-23396-1

**Published:** 2018-03-22

**Authors:** Mingjun Wu, Ruifan Zhang, Qing Zou, Yaoyao Chen, Min Zhou, Xingjie Li, Ran Ran, Qiang Chen

**Affiliations:** 1 Research Center for Stem Cell and Regenerative Medicine, Sichuan Neo-life Stem Cell Biotech INC, Chengdu, Sichuan China; 2 Department of Ophthalmology, Sichuan Academy of Medical Sciences & Sichuan Provincial People’s Hospital, Chengdu, Sichuan China; 3 Center for Stem Cell Research & Application, Institute of Blood Transfusion, Chinese Academy of Medical Sciences and Peking Union Medical College, Chengdu, Sichuan China

## Abstract

Mesenchymal stem/stromal cells (MSCs) derived from placental tissue show great therapeutic potential and have been used in medical treatment, but the similarity and differences between the MSCs derived from various parts of the placenta remain unclear. In this study, we compared MSCs derived from different perinatal tissues, including the umbilical cord (UC), amniotic membrane (AM), chorionic plate (CP) and decidua parietalis (DP). Using human leukocyte antigen (HLA) typing and karyotype analysis, we found that the first three cell types were derived from the foetus, while the MSCs from the decidua parietalis were derived from the maternal portion of the placental tissue. Our results indicate that both foetal and maternal MSCs share a similar phenotype and multi-lineage differentiation potential, but foetal MSCs show a significantly higher expansion capacity than do maternal MSCs. Furthermore, MSCs from all sources showed significant differences in the levels of several paracrine factors.

## Introduction

Human placenta is well known to not only play a fundamental and essential role in foetal development, nutrition, and tolerance, but also function as a bank of MSCs. Placental tissue can be easily obtained as medical waste. Placenta-derived MSCs can be procured from this medical waste, free of invasive procedures such as adipose tissue collection, and there are no ethical controversies surrounding its use unlike the embryonic stem cells. Considering the complexity of the placenta, this tissue can be conceptually divided into the foetal side, consisting of the amnion, chorion and umbilical cord, and the maternal side, consisting of the decidua. Numerus reports have been published on the MSCs that originate from different parts of the placenta^1–11^. Many of the perinatal sources, including the amniotic membrane (AM), chorionic plate (CP), decidua parietalis (DP) and umbilical cord (UC), have advantages over adult sources such as BM in terms of their ease of availability, lack of donor site morbidity, naivety of cells, abundance of stem cells in tissues, and high capacity for proliferation^7,12,13^.

The placenta has been largely used to study MSCs, and several studies have already compared the features (phenotype and function) of MSCs isolated from different placental tissues^14–24^. However, the origin of MSCs derived from all sources (AM, CP, DP and UC) of the placenta have not been determined, and there is a lack of comprehensive comparisons between MSCs. Moreover, optimal sources for specific clinical applications remain to be identified^25^. The hypothesis that all MSCs, regardless of their origins, are identical in their quality and function ignores their differences in biology and potential therapeutic use, which cannot be defined and characterized by current methods *in vitro*^26^. MSCs are routinely defined *in vitro* by cell surface antigen expression and differentiation potential. These features are also known as the minimal MSC criteria proposed by the International Society for Cellular Therapies (ISCT)^27^. However, these minimal criteria are not specific for MSCs and cannot distinguish the connective tissue cells that share the same properties^28^. Cell-cell adhesion mediated by vascular cell adhesion protein 1 (VCAM-1) is known to be critical for T cell activation and leukocyte recruitment to the site of inflammation. Therefore, VCAM-1 plays an important role in evoking effective immune responses. VCAM-1 is also reported to be a biomarker for a subpopulation of chorionic villi-derived MSCs with unique immunosuppressive activity^12^. This finding suggests that a better understanding of the functional properties indicating the potential impact on future clinical applications may be achieved by identifying the molecular pathways and cytokine profiling of MSCs^19,29^.

In our study, we compared MSCs derived from the UC, AM, CP of foetal origin and the DP of maternal origin in the placenta to understand their similarities and differences. The morphology and immunophenotype (assessed by flow cytometry) were analysed. HLA typing and karyotype analysis were carried out to determine the origin of the MSCs. Growth kinetics were evaluated using the population doubling time (PDT) and CCK-8. Cytokine secretion function was quantitatively analysed using the enzyme-linked immunosorbent assay (ELISA) kit. Our data suggest that VCAM-1 could be used as a biomarker to determine the CP-derived MSCs.

## Results

### Identification of placenta-derived MSCs

**Figure 1:**
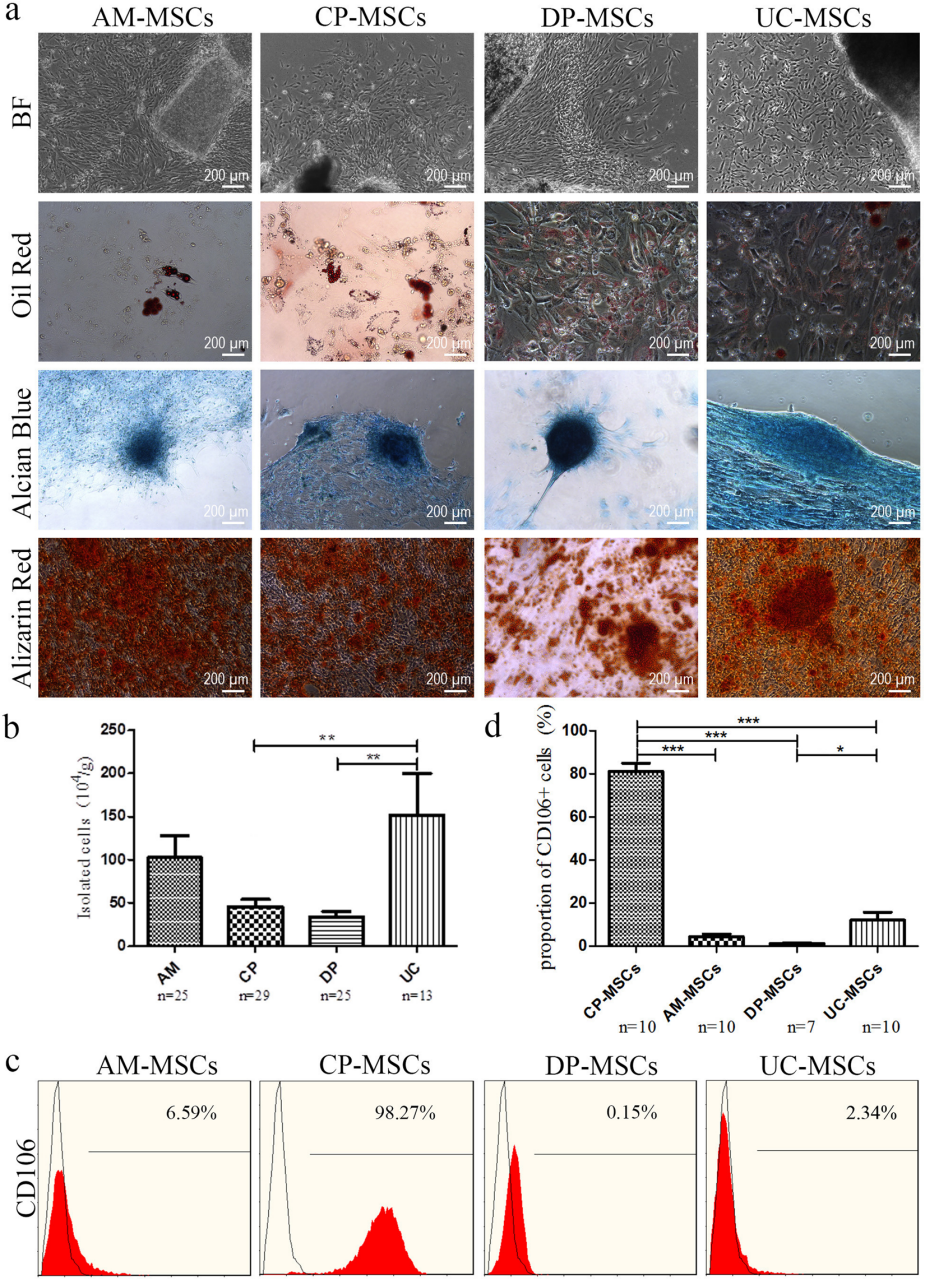
Characterization and isolation yield of different types of MSCs derived from perinatal tissues. (**a**) All MSCs exhibited a similar morphology and became positive for oil red O (adipocytic differentiation), alcian blue (chondrocytic differentiation), and alizarin red (osteocytic differentiation). (**b**) Original raw material, MSC isolation yield. Data are presented as the mean ± SEM (*p < 0.05, **p < 0.005). (**c**) Flow cytometric analysis of CD106 expression in different MSCs. (**d**) Statistical result of CD106 expression in different MSCs. Data are presented as the mean ± SEM (***P < 0.0001).

According to the ISCT criteria, the MSCs derived from AM, CP, DP and UC (Supplementary Fig. S1a,b) exhibited typical fibroblastoid, spindle-shaped morphology and displayed a high capacity to adhere to plastic when maintained in standard culture conditions using tissue culture flasks (Fig. 1a, top panel). There were significant differences in the cell isolation rates from different sources, ranging from 0.34 to 1.52 million single cells per gram tissue (Fig. 1b). According to our data, MSCs cultured from all sources could be established with a comparable positive rate.

After 21 days of induction with the respective induction media, AM-MSCs underwent low-level trilineage differentiation. In contrast, the three other types of MSCs showed relatively higher differentiation potential (Fig. 1a). CP-, DP-, and UC-MSCs from all three donors differentiated into all three induced lineages (adipocytes, osteoblasts and chondroblasts). AM-MSCs from donors 1 and 2 showed only adipogenic and osteogenic differentiation potential, and only donor 3 showed trilineage differentiation potential (Supplementary Fig. S2).

To determine the most significant differences among these MSCs, we compared the phenotypes of MSCs isolated from the human placenta using identical methods. Each type of MSC was tested in 10 donors. A series of cell markers was examined at passage 3 of *in vitro* cultivation, including the classical MSC phenotypes as defined by the ISCT criteria (CD14, CD34, CD45, CD73, CD90, CD105 and HLA-DR), embryonic stem cell markers (SOX2 and SSEA4) and VCAM-1, also known as CD106. AM-, CP-, DP- and UC-MSCs showed similar expression levels of MSC-specific surface markers (CD73, CD90 and CD105) and an absence of leucocyte, haematopoietic cell, or monocyte/macrophage markers (CD45, HLA-DR, CD34 and CD14) (Supplementary Fig. S3). All of these MSCs highly expressed the SOX2 and SSEA4 embryonic stem cell markers, as well as mesenchymal markers, including CD73, CD90 and CD105 (Supplementary Fig. S4). The most significant difference in their phenotype was the expression of CD106, which was expressed highly in CP-MSCs (81.10 ± 12.28%), moderately in UC-MSCs (12.07 ± 11.43%), and slightly in AM-MSCs (4.27 ± 4.39%). DP-MSCs did not express CD106 (Fig. 1c,d).

### Origin determination

HLA analysis of the culture-expanded cells from the same placental sample (n = 3) showed that AM-, CP- and UC-derived MSCs were of foetal origin, and DP-derived MSCs were of maternal origin (Table 1). However, some of the culture-expanded DP-derived cell populations expressed both foetal- and maternal-specific alleles (data not shown).

**Table 1:** HLA typing of culture-expanded MSCs from the same placental sample.

Donor	Genotype	AM-MSCs	CP-MSCs	DP-MSCs	UC-MSCs
1	HLA-A	A24, -	A24, -	A11, A24	A24, -
	HLA-B	B39, B60	B39, B60	B13, B39	B39, B60
	HLA-DRB1	DR12, DR13	DR12, DR13	DR12, DR15	DR12, DR13
2	HLA-A	A2, A24	A2, A24	A24, -	A2, A24
	HLA-B	B75, B61	B75, B61	B8, B61	B75, B61
	HLA-DRB1	DR12, DR15	DR12, DR15	DR17, DR12	DR12, DR15
3	HLA-A	A2, A33	A2, A33	A2, -	A2, A33
	HLA-B	B13, B61	B13, B61	B13, B46	B13, B61
	HLA-DRB1	DR14, DR15	DR14, DR15	DR8, DR15	DR14, DR15

**Figure 2:**
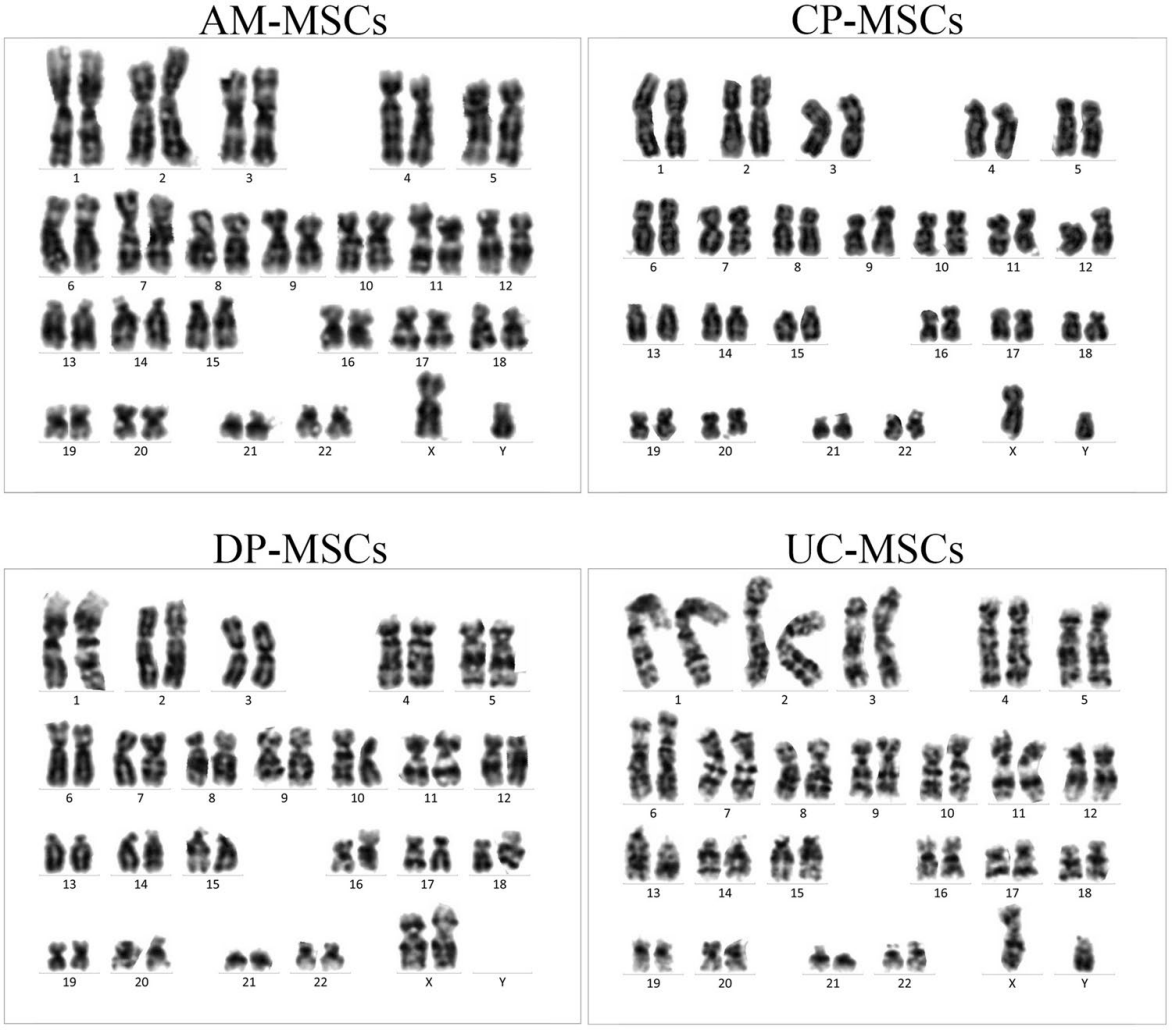
Karyotype analysis of different MSCs derived from different sources of the placenta of male babies (n = 3). G-band staining revealed that AM-, CP- and UC-MSCs were foetal cells exhibiting a normal 46, XY karyotype, and DP-MSCs were maternal cells exhibiting a normal 46, XX karyotype.

To confirm that these MSCs in culture were derived from the foetal or maternal placenta, the cytogenetic karyotypes of the cells from the same placenta (n = 4) of male babies were analysed. XX sex chromosomes were detected in DP-MSCs, and XY chromosomes were detected in AM-, CP- and UC-MSCs (Fig. 2).

### Growth characteristics

**Figure 3:**
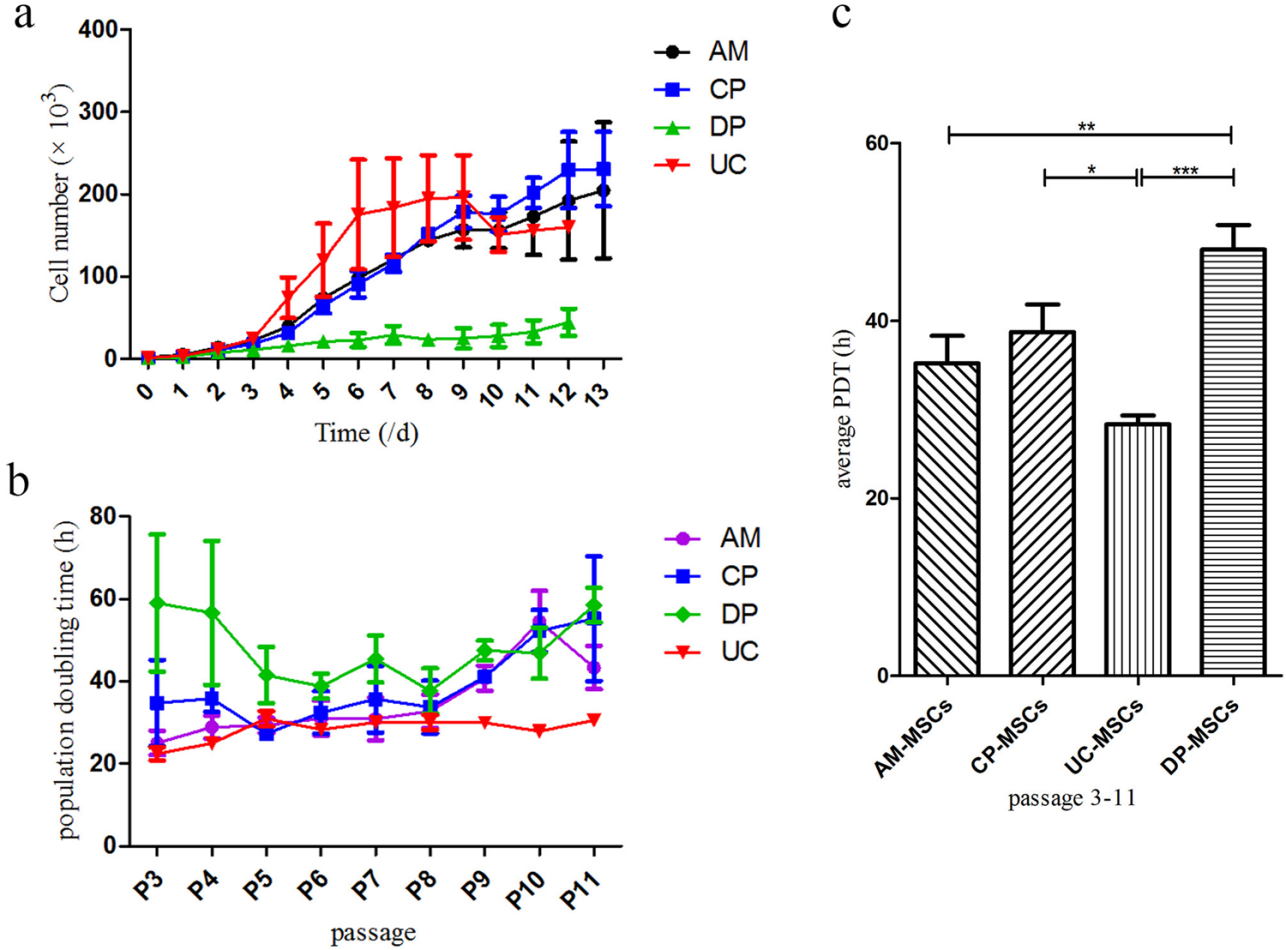
Proliferative potential of different sources of MSCs. The number of MSCs was counted each time following subculture from passages 3 to 11 (n = 3 donors). (**a**) Growth curves of different types of MSCs. (**b**) The population doubling time was also calculated based on cell counts. (**c**) Comparison of average population doubling time of different sources of MSCs following subculture from passages 3 to 11. Data are presented as the mean ± SEM (*P < 0.05. **P < 0.005. ***P < 0.0001).

The growth curves of all MSCs show that the DP-MSCs grew the slowest (Fig. 3a). During cell proliferation, the MSCs were cultured up to passage 11. Based on our calculations of the cell population doubling time, the cell PDT of the UC-MSCs was 28.34 ± 2.89 h, and that of the AM-, CP- and DP-MSCs was 35.19 ± 9.28 h, 38.71 ± 9.27 h and 48.01 ± 8.26 h, respectively (Fig. 3b,c). Thus, the order of the growth rate of the cells was as follows (from the fastest to the slowest): UC-, AM-, CP- and DP-MSCs.

### Secretion patterns of selected growth factors and cytokines

**Figure 4:**
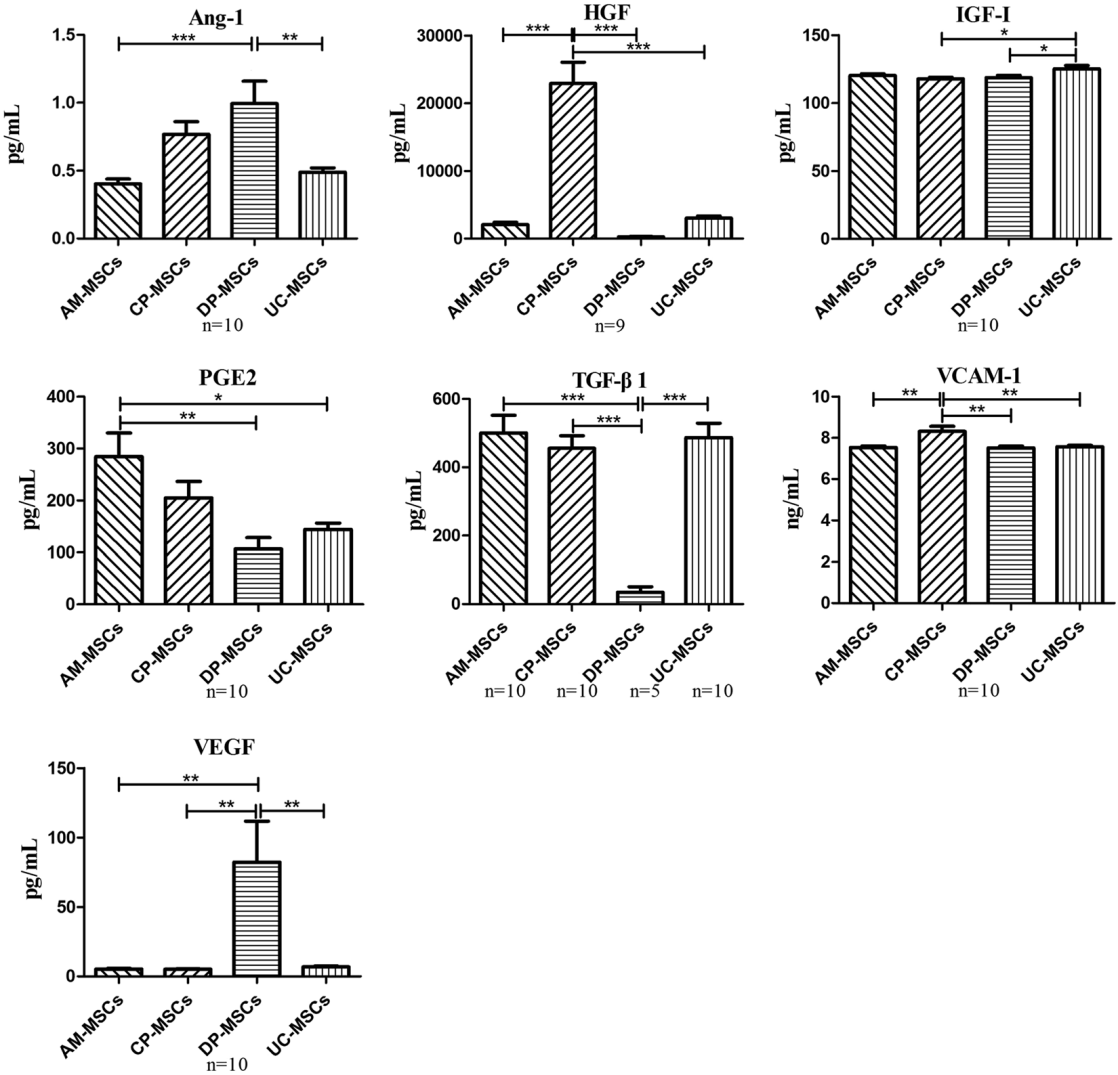
Comparison of the secretion patterns of selected growth factors and cytokines. Differences in the four sources were determined to be significant and were labelled with a star if the *P*-value determined using ANOVA followed by Tukey’s test was <0.05. Data are expressed as the mean ± SEM (*P < 0.05. **P < 0.005. ***P < 0.0001). Ang-1, angiopoietin-1; HGF, hepatocyte growth factor; IGF-I, insulin-like growth factor I; PGE2, prostaglandin E2; TGF-β1, transforming growth factor beta 1; VCAM-1, vascular cell adhesion molecule-1; VEGF, vascular endothelial growth factor.

Secretion of paracrine factors, including human angiopoietin-1 (Ang-1), hepatocyte growth factor (HGF), insulin-like growth factor I (IGF-I), prostaglandin E2 (PGE2), transforming growth factor beta 1 (TGF-β1), VCAM-1 and vascular endothelial growth factor (VEGF), in all MSCs was assessed using ELISA kits according to the manufacturer’s instructions. MSCs from all sources showed significant differences in the levels of selected factors. AM-MSCs showed the highest secretion of PGE2 and TGF-β1. CP-MSCs showed the highest secretion of HGF and VCAM-1. DP-MSCs showed the highest secretion of Ang-1 and VEGF and the lowest secretion of TGF-β1, while UC-MSCs showed the highest secretion of IGF-I (Fig. 4).

## Discussion

In this study, we performed a side-by-side comparison of 4 populations of MSCs derived from perinatal tissues, including AM, CP, DP and UC. In summary, this study resulted in the following major conclusions:

First, we analysed the origin of different perinatal tissue-derived MSCs. HLA typing and karyotype analysis confirmed that AM-, CP- and UC-derived MSCs were of foetal origin, and DP-derived MSCs were of maternal origin. Moreover, we observed significant differences in the proliferative potential among the 4 populations of MSCs, and the proliferation rate from the fastest to the slowest was as follows: UC-, AM-, CP- and DP-MSCs. The growth curve showed that the proliferative capacity of the MSCs of foetal origin was significantly greater than that of the MSCs of maternal origin.

Second, we found that MSCs derived from different perinatal tissues are not identical in terms of their biological properties. Although MSCs from all sources were shown to express similar surface markers according to the ISCT criteria and some pluripotency related markers; for instance, SOX2 and SSEA4, CP-MSCs show the highest CD106 expression compared to the other three MSCs, which displays a positive correlation with the immunosuppressive effect. CD106 is known to play an important role in embryonic development in the formation of the umbilical cord and placenta^30^. Moreover, surface molecules, such as CD106 and CD54, are considered to be important for the immunomodulation of MSCs^31^.

Third, MSCs derived from different tissues have been demonstrated in numerous studies to differentiate into cells in the mesodermal lineage, such as adipocytes, osteoblasts and chondroblasts^32–35^. Our results demonstrated that there are quantitative differences between various populations of MSCs derived from different perinatal tissues with respect to their differentiation potential. Our data indicated that AM-MSCs underwent trilineage differentiation at a low level. Furthermore, the differentiation potential of foetal (AM origin) vs. adult (DP origin) MSCs in our work showed that the proliferative capacity of the adult (maternal) cells was significantly lower than that of the feotal cells which is inconsistent with their differentiation potential (Fig. 3, supplementary Fig. S2).

Fourth, the secretion patterns of selected growth factors and cytokines revealed that MSCs from all sources showed distinct differences in the levels of the selected factors. These factors were selected because multiple studies have shown that they are secreted by MSCs during inhibition of apoptosis, immunomodulation, anti-fibrotic processes, angiogenesis, chemotaxis and haematopoiesis induction/support *in vitro* or *in vivo*^36–41^. Recent studies have demonstrated that the high expression of HGF and VCAM-1 in MSCs was associated with a favourable angiogenic potency and displayed therapeutic efficacy in hindlimb ischaemia^42,43^.

In conclusion, our study compared MSCs derived from different perinatal tissues to better understand the similarities and differences among these cell types. The origin and purity of each cells was confirmed by HLA typing and karyotype analysis and showed that the first three cell types were of foetal origin and the last cell type was of maternal origin from the placental tissue. Although both foetal and maternal MSCs have similar phenotypes and multi-lineage differentiation potential, foetal MSCs showed a significantly higher expansion capacity than did maternal MSCs, and furthermore, MSCs from all sources showed significant differences in the levels of selected paracrine factors. These findings may offer clues to the clinical application of different types of MSCs. For instance, AM-MSCs may be used in the treatment of premature ovarian ageing due to their higher secretion of PGE2 and TGF-β1^44^; CP-MSCs display potential pro-angiogenic activity due to the higher secretion of HGF and VCAM-1^43^ and could be used in angiogenic therapy; and DP-MSCs show advantages in the treatment of critical limb ischaemia because of the higher secretion of VEGF and Ang-1^45^. Compared to AM-, CP- and DP-MSCs, UC-MSCs secreted higher levels of a wide range of selected paracrine factors. Thus, UC-MSCs may be a source of cell therapy to treat other diseases. Furthermore, it would be necessary to identify the ability of MSCs derived from different sources differentiating into various types of cells specifically into the three germ layers such as ectoderm (epithelial and neuronal cells), mesoderm (endothelial cells and cardiomyocytes) and endoderm (hepatocytes and insulin producing β-cells). More functional studies are required to confirm these findings and to obtain a further understanding of the biological differences of MSCs from various sources so that the most suitable MSCs for treatment of specific diseases can be verified and acquired.

## Methods

### Isolation and culture of MSCs from the human placenta and umbilical cord

The experiments involving human tissue were approved by the Research Center for Stem Cell and Regenerative Medicine, Sichuan Neo-life Stem Cell Biotech INC./ Center for Stem Cell Research & Application, Institute of Blood Transfusion, Chinese Academy of Medical Sciences and Peking Union Medical College (CAMS & PUMC). All the experiments were carried out in accordance with the approved guidelines. Human placentae (n = 60) and umbilical cords (n = 13) were collected from healthy, full-term, uncomplicated pregnancies. Written informed consent was obtained from the mothers and the donors.

First, UCs were dissected longitudinally, and the arteries and veins were removed. The remaining pieces were chopped mechanically. Second, the decidua parietalis attached to the maternal side of the human placenta was manually separated from the chorion. Third, the placental amnion attached to the foetal side of the human placenta was separated from the chorionic plate. Finally, the chorionic plate without the amnion and decidua basalis was separated from the human placenta. All of the above three tissues were washed thoroughly with phosphate-buffered saline (PBS; pH 7.4) to remove excess blood. The tissues were rinsed in PBS and were extensively minced. All of the explants, including the UCs, were transferred into 100 mm plates (Corning, USA). Complete culture medium (Dulbecco’s modified Eagle’s medium/nutrient mixture F-12, DMEM-F12 containing 10% foetal bovine serum, 100 mg/mL streptomycin and 100 U/mL penicillin) was added to the plates, and the explants were cultured at 37 °C in a 5% CO_2_ incubator and left undisturbed to allow the cells to migrate from the explants. After 10–15 days, MSC-like cells were found around the fragments. MSCs were identified on the basis of their fibroblastic morphology and phenotypic characterization, which was performed after passage 3, and were used in subsequent experiments. The cell cultures at different time intervals were observed under an inverted phase contrast microscope (Leica DMI3000 B, Leica Microsystems Inc., Germany) and the images were captured using Leica Application Suite Version 3.8.0 software.

### Determination of the maternal and foetal origin of MSCs

To analyse the origin of culture-expanded MSCs derived from the amnion, chorionic plate, decidua parietalis and umbilical cord, molecular HLA typing was performed on DNA obtained from expanded MSCs using PCR-SSP with an AllSet+ Gold SSP HLA-A\B\DRB1 kit (ONE LAMBDA, Canoga Park, CA).

### Flow cytometry analysis

For phenotypic identification of the MSCs derived from all sources, a total of 1 × 10^6^ cells were divided into aliquots in 1.5 mL microcentrifuge tubes, and the samples were centrifuged at 500 × g for 5 minutes. Pelleted cells were washed twice in phosphate-buffered saline (PBS) supplemented with 0.2% foetal bovine serum (FBS) (Gibco, Life Technologies, USA). The cells were then suspended in 50 *μ*L of PBS with 1% bovine serum albumin (BSA), and the following cell surface epitopes were detected: anti-human CD73-PE, CD90-FITC, CD105-PE, VCAM-1-PE, CD166-PE, CD14-PE, CD34-PE, CD45-Pc7, HLA-DR-FITC (BD Biosciences, USA), SOX2-PE and SSEA4-PE (eBioscience, USA). Appropriate isotype controls were used for each antibody to assess for nonspecific antibody binding. The cells were then analysed using a flow cytometry instrument (FC500; Beckman Coulter, USA) and data processing software (FlowJo 10.0.7; TreeStar, USA).

### Growth kinetics analysis

The proliferation of MSCs from P3 to P11 was assessed (n = 3). MSCs from all sources were inoculated on a six-well culture plate at a density of 7–10 × 10^5^ cells/well, and the cells were counted until they reached 100% confluency. The PDT was calculated using the following formula:

$${\rm{PDT}}=({\rm{CT}}\times \,\mathrm{ln}\,2)/\,\mathrm{ln}({{\rm{N}}}_{{\rm{f}}}/{\rm{Ni}}),$$

where CT is the cell culture time, Ni is the initial number of cells, and N_f_ is the final number of cells^46^.

### Proliferation assay

The proliferation of MSCs from all sources was determined using the Cell Counting Kit-8 (CCK-8, Dojindo Molecular Technology, Japan). MSCs were plated at a density of 2,000 cells per well in 96-well plates in standard culture medium. After 4 hours of incubation, 10 µL of CCK-8 was added to each well, and the plates were incubated at 37 °C. Optical density (OD) was measured every 24 hours with a spectrophotometer (*Multiskan GO*, Thermo Scientific) at 450 nm. Cell viability was calculated relative to the control.

### *In vitro* differentiation assay for MSCs

AM-, CP-, DP- and UC-derived MSCs were differentiated into adipocytes, osteoblasts and chondrocytes after three passages as follows. In brief, for adipogenic, osteogenic or chondrogenic differentiation, MSCs from all sources were seeded into 12-well plates at 200,000 cells per well and were maintained in standard culture medium until confluency. Cells were exposed to adipogenic, osteogenic or chondrogenic induction medium (All from Gibco, Life Technologies, Grand Island, USA) for 21 days. Cells were fixed in 4% paraformaldehyde. To assess adipogenic differentiation, lipid droplets of differentiated cells were stained using oil red O. To assess osteogenic differentiation, cells were stained with alizarin red S. To assess chondrogenic differentiation, cells were stained with alcian blue. Control cells were maintained in standard culture medium over the same time period (All stains were procured from Sigma Aldrich, St Louis, USA). The stained plates were observed under an inverted phase contrast microscope (Leica DMI3000 B, Leica Microsystems Inc., Germany) and the images were captured using Leica Application Suite Version 3.8.0 software.

### Karyotype analysis

To analyse the karyotype of the AM-, CP-, DP- and UC-derived MSCs from the same placenta (male new-born), cell division was blocked at metaphase with 0.1 μg/mL colcemid (Calbiochem, Germany) for 2 hours at 37 °C. The cells were washed and trypsinized, resuspended in 0.075 M KCl, incubated for 20 minutes at 37 °C, and fixed with methanol and acetic acid (3:1). G band standard staining was used to visualize the chromosomes. At least 20 metaphase-nuclei were detected in each sample. The cells in metaphase were analysed and reported on by a certified cytogenetic laboratory according to the International System for Human Cytogenetic Nomenclature.

### Quantification of secreted factors

Culture supernatants were generated as follows. Cells were seeded in standard culture medium at a density of 10,000 cells/cm^2^. After 72 hours, cell-free supernatants were collected and were stored at −80 °C. The levels of hepatocyte growth factor (HGF), angiopoietin-1 (Ang-1), vascular endothelial growth factor (VEGF), vascular cell adhesion molecule-1 (VCAM-1), insulin-like growth factor I (IGF-I), prostaglandin E2 (PGE2) and transforming growth factor beta 1 (TGF-β1) were measured using the respective ELISA kit (Bio-Rad) according to the manufacturer’s protocol.

### Statistical analysis

Statistical analyses were performed using GraphPad Prism version 5.0 (California, USA). Comparisons of parameters for more than three groups were made by one-way analysis of variance (ANOVA) followed by Tukey’s test. Parametric data are expressed as the means ± standard deviation (SD). A value of *P* < 0.05 was considered statistically significant.

## Electronic supplementary material

Supplementary Information
