# Supplementary material for: Comparison of the Biological Characteristics of Mesenchymal Stem Cells Derived from the Human Placenta and Umbilical Cord
Source: Sci Rep. 2018 Mar 22;8:5014. doi: 10.1038/s41598-018-23396-1 (PMC5864926; doi:10.1038/s41598-018-23396-1)
Supplement: Supplementary file 1 — Supplementary Information [file 41598_2018_23396_MOESM1_ESM.pdf]

Comparison of the Biological Characteristics of Mesenchymal Stem Cells Derived from the Human Placenta and Umbilical Cord

Mingjun Wu,[1] Ruifan Zhang,[2] Qing Zou,[1] Yaoyao Chen,[1] Min Zhou,[1] Xingjie Li,[1] Ran Ran[1] & Qiang Chen[1,3]

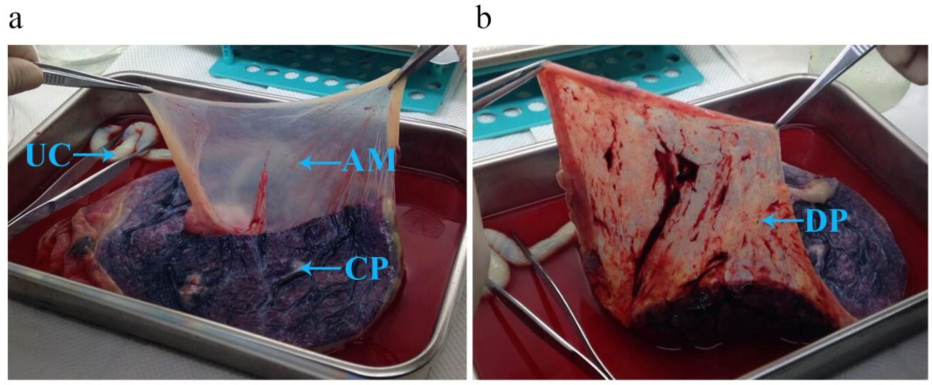

Figure S1. Perinatal tissues including (a) Umbilical cord (UC), amniotic membrane (AM), chorionic plate (CP) and (b) Decidua parietalis (DP).

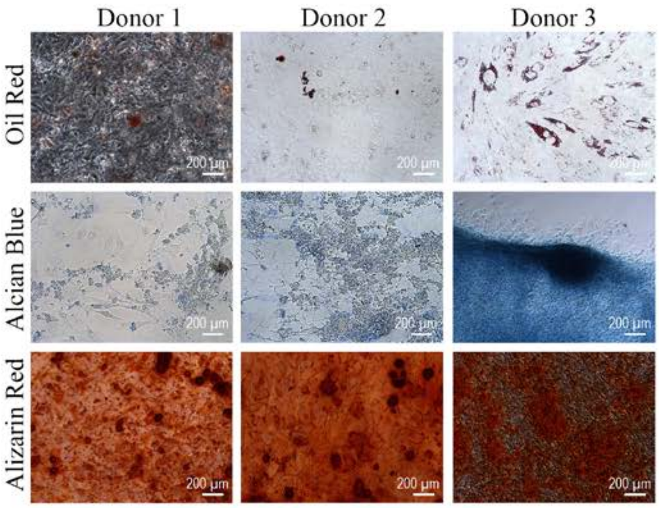

Figure S2. Trilineage differentiation potential of AM-MSCs (n=3). Related to Figure 1.

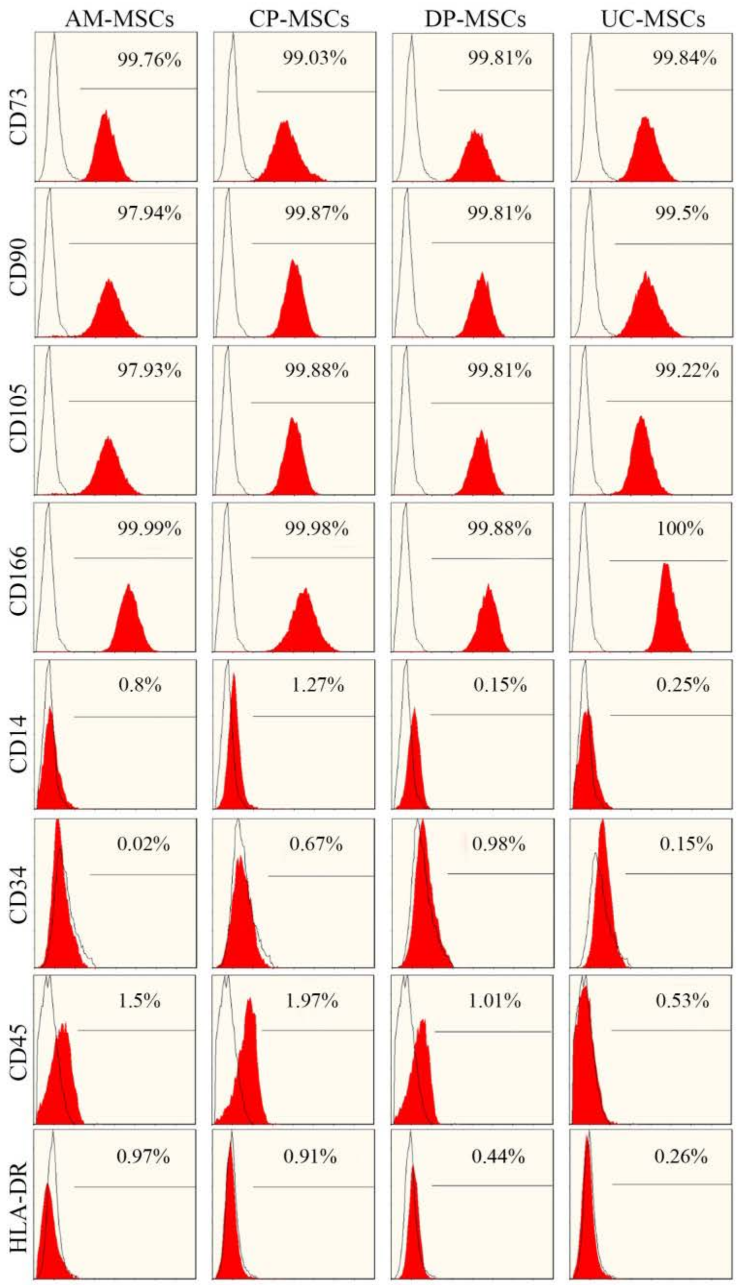

Figure S3. Flow cytometric analysis of typical MSCs markers. Related to Figure 1.

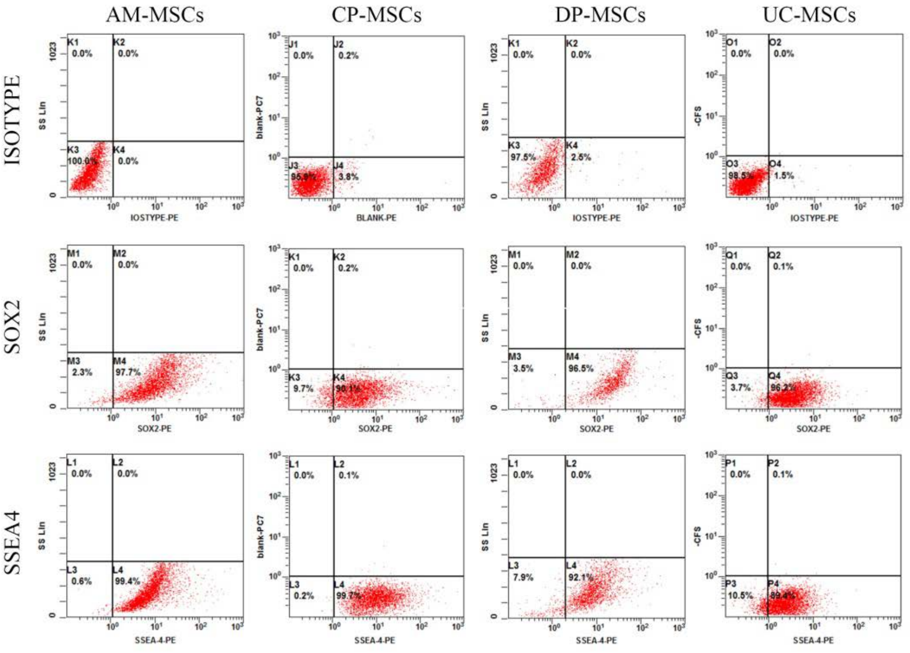

Figure S4. Flow cytometric analysis of embryonic stem cell markers. Related to Figure 1.
